# Scleroderma in a Patient on Capecitabine: Is this a Variant of Hand-Foot Syndrome?

**DOI:** 10.7759/cureus.663

**Published:** 2016-06-30

**Authors:** Muhammad W Saif, Archana Agarwal, James Hellinger, Dorothy J Park, Elizabeth Volkmann

**Affiliations:** 1 Hematology/Oncology, Tufts Medical Center; 2 Medicine, Tufts Medical Center; 3 Oncology, Cedars-Sinai Medical Center; 4 Medicine, UCLA

**Keywords:** capecitabine, colon cancer, breast cancer, rash, scleroderma

## Abstract

Drug-induced scleroderma is a rare adverse effect of some chemotherapeutic drugs, such as taxanes and bleomycin. Capecitabine, an oral fluoropyrimidine approved for the treatment of metastatic breast and colon cancer, commonly causes cutaneous side effects including the hand-and-foot syndrome (HFS). Scleroderma-like skin changes associated with HFS associated with capecitabine is rare. However, diffuse scleroderma has never before been reported. We report a case of capecitabine-induced diffuse/systemic scleroderma in an 86-year-old female treated with capecitabine for metastatic colorectal cancer. She developed progressive skin and visceral sclerosis involving the lungs. We discuss the association between chemotherapy and scleroderma. We believe this is the first case of diffuse/systemic capecitabine-induced scleroderma without the presence of HFS. Early diagnosis is essential as fibrosis might be prevented in early stages. The capecitabine should be discontinued as early as possible.

## Introduction

Capecitabine is an orally-administered fluoropyrimidine carbamate that has currently been approved for treatment of metastatic breast and colon cancer [1]. It is converted to its active metabolite 5-fluorouracil (5-FU) by thymidine phosphorylase [2], which is located primarily in the liver and tumor tissue. This local release of the active metabolite causes less toxicity than 5-FU. The dose-limiting side effects of capecitabine include diarrhea, hyperbilirubinemia, and hand-and-foot syndrome [1-2].

Hand-and-foot syndrome (HFS), also known as palmar-plantar erythrodysesthesia (PPE), is the most frequently reported side effect of capecitabine therapy [3]. Patients can present with various degrees of dysesthesia, painful erythema, and edema of the palms of the hand and soles of the feet, followed by desquamation. A grading system for HFS was proposed based on both functional and clinical status and divided into three grades:

Grade 1 consists of numbness, dysesthesia/paresthesia, tingling, painless swelling, or erythema not disrupting normal activities.

Grade 2 manifests as painful erythema with swelling that disrupts daily activities.

Grade 3 consists of desquamation, ulceration, blistering, severe pain, or any symptoms leading to an inability to perform daily activities.

However, the manifestations of HFS in dark skinned patients could be different as we previously described and may consist of hyperpigmentation and gradual thickening of the palms, fingers, and soles [4]. On the other hand, only two case reports of scleroderma-like skin changes (cutaneous) associated with HFS have previously been reported [5-6]. Here, we report a case of diffuse drug-induced scleroderma induced by capecitabine without the evidence of manifestations of severe HFS.

## Case presentation

An 86-year-old caucasian woman presented with Stage-IV colorectal cancer. The patient agreed to participate and was explained the nature and objectives of this study, and informed consent was formally obtained. No reference to the patient's identity was made at any stage during data analysis or in the report. The patient had a large, undifferentiated tumor at the splenic flexure along with liver metastasis. She underwent resection of the primary tumor due to an impending obstructing tumor. Because of her advanced age, it was decided to proceed with monotherapy consisting of capecitabine only. A pre-treatment genetic testing for capecitabine consisting of thymidine synthase (TYMS) and dihydropyrimidine dehydrogenase (DPD/DPYD) analysis was performed that showed intermediate risk for 5-FU and related agents' toxicity.Therefore, capecitabine was initiated at a low dose (500 mg BID) and then gradually increased to a maximum dose of 1500 mg/m^2^/day for two-weeks-on and one-week-off schedule days = 1 cycle). She tolerated it well except mild intermittent diarrhea (Grade 1) and Grade 1 HFS manifested as erythroderma on the palms of both hands and later some dry desquamation of limited duration which promptly resolved when capecitabine was held. Her restaging imaging including a PET-CT scan showed a good response in the liver metastasis. All of her symptoms resolved when capecitabine was discontinued.

After a few months of treatment with capecitabine, she noticed 'dry skin with itching' had developed for which she saw a dermatologist. Subsequently, she also developed a progressive cough and shortness of breath, hoarseness, mild hypertension, and symptoms of gastroesophageal reflux disease (GERD) manifested as heartburn with reflux symptoms. During that period, she was evaluated by a multi-disciplinary team comprising of a pulmonologist, rheumatologist, ear-nose-throat (ENT), and gastroenterology in addition to her oncologist. Pulmonary fibrosis was noticed on the CT scan of the chest. In addition, she also developed a new onset renal dysfunction during that period.

Anti-nuclear, anti-centromere, antibodies to Smith antigen, antibodies to ribonucleoprotein, and anti-scl 70 antibodies were all negative. However, complement fixing antinuclear antibodies (C-ANA) on HEP-2 cells showed higher titer (320) with speckled pattern and C-ANA/ANA titers ratio of 320/40. A C-ANA with such a pattern and C-ANA/ANA titers ratio is suggestive of systemic sclerosis or one of the other diseases in group A of systemic connective tissue diseases. Moreover, a skin biopsy was performed.  A biopsy with surface dimensions of 2.5 mm x 2 mm was reviewed from the upper right wrist skin lesion. For the C+DIF test, a fresh source of complement was added to the biopsy sections followed by fluorescein-labeled antibodies to the C3 component of the complement. This biopsy included skin of the epidermis and dermis with no structural deformities. Direct immunofluorescent studies of the skin biopsy specimen showed granular focal deposits of fibrin at the dermal-epidermal junction and deposits of IgG, IgA, IgM, fibrin, and trace C3 in the connective tissue fibers to the epidermal nuclei. These positive C+DIF reactions and serum results are consistent with or at least suggestive of systemic scleroderma or another 'group A' systemic connective tissue disease.

She was placed on nifedipine, aspirin, lisinopril, and omeprazole. She was also started on cyclophosphamide treatment at the starting dose of 500 mg and then dose escalated to 700 mg and finally to 900 mg as tolerated per her Rheumatologist's recommendation [7]. She did not tolerate the 900 mg of the third cycle; therefore, the dose was decreased back down to 700 mg, and she completed six cycles of treatment. Cyclophosphamide was stopped and replaced with mycophenolate mofetil (MMF) at a dose of 0.5 g per day to a maximum of 2 g per day [8]. Her dyspnea resolved with treatment with mycophenolate. Her lung function also improved and eventually normalized. She was tapered off of mycophenolate after 23 months of therapy. Six months after cessation of mycophenolate, her lung function remained stable.

## Discussion

To our knowledge, our patient is the first case report of diffuse scleroderma in a patient treated with capecitabine without concomitant HFS. Two other case reports have described scleroderma-like skin changes associated with HFS [5-6]. The first case report described a 70-year-old male with Stage 4 signet ring carcinoma of the stomach treated with capecitabine at a dose of 2500 mg/m^2^ [4]. The patient developed hyperpigmentation of the palms, fingers, and soles followed by scleroderma-like skin changes. Biopsy revealed diffuse dermal sclerosis. Anti-nuclear, anti-centromere, anti-scl 70 antibodies, and rheumatoid factor were all negative. The second case described a 56-year-old male with unresectable adenocarcinoma of the sigmoid colon with hepatic metastasis who was treated with oral capecitabine (2000 mg twice daily) [6]. The patient developed HFS with scleroderma-like skin changes. The biopsy revealed dermal sclerosis with thick homogeneous eosinophilic collagen bundles. Both of the cases did not report any visceral involvement. Our patient developed diffuse sclerosis of her skin along with involvement of her lungs, kidneys, and esophagus without the development of HFS. Pulmonary fibrosis was noticed on the CT scan of the chest. She also developed new onset renal dysfunction during that period. Her laboratory data was significant for C-ANA with a speckled pattern and C-ANA/ANA titers ratio both suggestive of systemic sclerosis. Moreover, direct immunofluorescent studies of the skin biopsy specimen showed granular focal deposits of fibrin at the dermal-epidermal junction and deposits of IgG, IgA, IgM, fibrin, and trace C3 in the connective tissue fibers to the epidermal nuclei consistent with or at least suggestive of systemic scleroderma or another 'group A' systemic connective tissue disease. Based on these findings, capecitabine-induced systemic scleroderma was considered as the most probable diagnosis. 

Differential diagnoses include idiopathic, paraneoplastic, or other primary condition of scleroderma. One can argue that her development of systemic sclerosis might have been attributable to her underlying malignancy (paraneoplastic process) in addition to the drug effect especially when systemic sclerosis develops in older individuals (>70 years), such as this patient, and it is often preceded or followed by the development of a malignancy. We argue that this is a case of capecitabine-induced systemic scleroderma as opposed to paraneoplastic or primary condition of scleroderma because 1) development of scleroderma correlated to the onset of treatment with capecitabine, 2) serology results, 3) the findings on the direct immunofluorescent studies of the skin biopsy specimen, and 4) the patient’s underlying tumor was in remission with progression of sclerosis, thus ruling out paraneoplastic scleroderma.

The pathophysiology of HFS is not clearly understood. Researchers have suggested the involvement of the epidermis of the palms and soles, damage to the epithelial cells of the eccrine ducts, or damage to basal keratinocytes [2]. Our group has tried to investigate the effect of metabolizing enzymes in the metabolism of capecitabine and found no significant association of PPE with either higher tumor thymidine phosphorylase (TP) or lower tumor dihydropyrimidine dehydrogenase (DPD) levels [9]. Interestingly, this patient for her age was given genetic tests for DPYD and TYMS and was found to be at moderate risk. Whether this placed at any predisposition is an interesting thought as we have published atypical skin toxicities in patients with DPD deficiency following fluoropyrimidines.

Chemotherapy-induced scleroderma has been described with different chemotherapeutic agents including docetaxel, paclitaxel, bleomycin, uracil-tegafur, gemcitabine, and combination therapy with doxorubicin and cyclophosphamide. Taxane-induced scleroderma (both docetaxel and paclitaxel) usually begins with edema of the limbs followed by thickening of the skin [10]. Sclerosis is mostly seen in the lower extremities, although a few cases of diffuse scleroderma have been reported. Taxane-induced scleroderma is usually dose dependent. Most patients do not have classical clinical features of scleroderma such as nail-fold changes and Raynaud’s phenomenon. Visceral involvement is rare. Laboratory abnormalities associated with classical scleroderma such as anti-nuclear antibody, anti-topo-I antibody, and anti-centromere antibody are usually absent. Mild improvement to complete resolution can be seen upon discontinuation of the offending drug. However, some cases show no improvement. This could possibly be due to lack of early omission of the drug. The biopsy showed dermal sclerosis with collagen bundles and mononuclear cell infiltration similar to classical scleroderma. Bleomycin-induced scleroderma has similar features. Most patients have limited-type cutaneous sclerosis. Raynaud’s phenomenon, visceral involvement, and immunologic abnormalities are usually absent. Some cases show improvement upon discontinuation of the drug. On the other hand, doxorubicin and cyclophosphamide-induced scleroderma mostly present with Raynaud’s phenomenon and diffuse sclerosis, which do not resolve with discontinuation of the drug or treatment with steroids. Immunologic testing is, however, negative, no end-organ damage has been reported in the literature, and the biopsy is consistent with systemic sclerosis. Uracil-Tegafur and gemcitabine-induced scleroderma present with limited cutaneous sclerosis.

Treatment regimens for systemic sclerosis depend on the presentation of the disease and complexity of the symptoms. ACE or angiotensin II inhibitors are generally used in patients with renal involvement while proton pump inhibitors or H2 blockers can alleviate symptoms associated with GI involvement. Calcium-channel blockers, prostaglandins, and cyclophosphamide have been tested in patients with pulmonary disease and showed variable results. Mycophenolate mofetil has also been used in patients with diffuse progressive cutaneous systemic sclerosis. Both mycophenolate and cyclophosphamide have each been demonstrated in randomized controlled trials to improve lung function and dyspnea in patients with systemic sclerosis-related interstitial lung disease [7-8].

## Conclusions

In summary, if similar cases of scleroderma continue to be recognized in patients receiving capecitabine, then this drug should be included in the potential etiology. The actual pathophysiology is not known at present, but it is possible that chemotherapy-induced scleroderma is different from classical scleroderma in many ways as described above. Early diagnosis is essential as fibrosis might be prevented in early stages. The offending drug should be discontinued as early as possible.
